# Radiation Dose to the Eye Lens through Radiological Imaging Procedures at the Surgical Workplace during Trauma Surgery

**DOI:** 10.3390/ijerph16203850

**Published:** 2019-10-11

**Authors:** Christian Apelmann, Birgitt Kowald, Nils Weinrich, Jens Dischinger, Albert Nienhaus, Klaus Seide, Heiko Martens, Christian Jürgens

**Affiliations:** 1 BG Klinikum Hamburg, Bergedorfer Str. 10, 21033 Hamburg, Germany; 2 Northern German Seminar for Radiation Protection gGmbH at the Christian-Albrechts-University Kiel, 24098 Kiel, Germany; 3 Competence Centre for Epidemiology and Health Services Research for Healthcare Professionals (CVcare), University Medical Centre Hamburg-Eppendorf (UKE), 20246 Hamburg, Germany; 4 B.A.D. Gesundheitsvorsorge und Sicherheitstechnik GmbH, 21033 Hamburg, Germany

**Keywords:** eye lens dose, eye dosimetry, fracture, C-arm X-ray, trauma surgeon, hand surgeon, surgical assistant, dosis area product

## Abstract

*Background*: Due to the drastic reduction of the eye lens dose limit from 150 mSv per year to 20 mSv per year since 2018, the prospective investigation of the estimated dose of the eye lens by radiological imaging procedures at the surgical site during trauma surgery in the daily work process was carried out. This was also necessary because, as experience shows, with changes in surgical techniques, there are also changes in the use of radiological procedures, and thus an up-to-date inventory can provide valuable information for the assessment of occupationally induced radiation exposure of surgical personnel under the current conditions. *Methods*: The eye lens radiation exposure was measured over three months for five trauma surgeons, four hand surgeons and four surgical assistants with personalized LPS-TLD-TD 07 partial body dosimeters Hp (0.07). A reference dosimeter was deposited at the surgery changing room. The dosimeters were sent to the LPS (Landesanstalt für Personendosimetrie und Strahlenschutzausbildung) measuring institute (National Institute for Personal Dosimetry and Radiation Protection Training, Berlin) for evaluation after 3 months. The duration of the operation, occupation (assistant, surgeon, etc.), type of surgery (procedure, diagnosis), designation of the X-ray unit, total duration of radiation exposure per operation and dose area product per operation were recorded. *Results*: Both the evaluation of the dosimeters by the trauma surgeons and the evaluation of the dosimeters by the hand surgeons and the surgical assistants revealed no significant radiation exposure of the eye lens in comparison to the respective measured reference dosimeters. *Conclusions*: Despite the drastic reduction of the eye lens dose limit from 150 mSv per year to 20 mSv per year, the limit for orthopedic, trauma and hand surgery operations is well below the limit in this setting.

## 1. Introduction

Directive 2013/59/EURATOM [1] of the Council of the European Union, published in 2014, provides for a significant reduction in the dose limit for the ophthalmic lens under occupational radiation exposure from 150 mSv/year to 20 mSv/year. The directive was transposed into national law in the form of the German Radiation Protection Law [2] in combination with the German Radiation Protection Ordinance [3] and thus the dose limit values of 20 mSv per year or 100 mSv in 5 years in occupationally exposed persons must not be exceeded, in the latter case 50 mSv per year may not be exceeded. The recommendations of the International Commission on Radiation Protection of 2012 [4] are hereby implemented. The background to this was radiobiological and epidemiological findings, which pointed out that the risk of cataracts after exposure to ionized radiation has been underestimated.

To date, the International Commission on Radiological Protection has assumed that cataract is clearly a deterministic effect with threshold doses of 0.5–2 Gy after acute exposure and 5–6 Gy after long-term exposure [5]. These previous thresholds were the basis for the previous limit of 150 mSv/year. However, more recent studies have shown that there are specific suspected cases below 100 mGy [6].

In recent years, a large number of radiological exposure studies have been published by radiological imaging procedures of surgical medical personnel [6,7,8,9,10,11,12,13,14,15,16,17,18,19,20,21,22,23,24,25,26,27,28,29,30,31,32,33,34,35,36,37,38,39,40,41,42,43,44,45,46,47,48]. The largest share was related to interventional cardiology and gastroenterology, while significantly fewer studies were found for orthopedic and trauma surgery [7]. So the data situation is limited to the examination of the radiation exposure of the eye lens dose during trauma/orthopedic interventions, especially against the background of the reduced limit of 20 mSv/year [12].

The first time, in 1977, Wahl and Even showed that critical levels of ocular radiation exposure are achieved with longer turn-on times during osteosynthesis, and they explicitly warned against frequent use for this reason [8]. Miller et al. described an average radiation exposure in the eye area of 0.2 mSv per operation with a mean fluoroscopic time of 330 seconds in 1983 [20].

By the end of the 1990s, Fuchs et al. [9,10,11] presented results of a prospective study on the radiographic exposure of the surgeon in 24 trauma operations (8-K wire osteosynthesis at the radius, 8 lumbar fixator internal (LWS) and eight intramedullary nail osteosynthesis on the femur) by perioperative radiographic imaging procedures. For the lens of the eye, a maximum radiation dose of 93.1 μSv was recorded for the treatment of the lumbar spine using an internal fixator. The authors came to the conclusion that even with a high frequency of operation for the surgeon, there is no danger of exceeding the statutory limit of 150 mSv per year.

In 2005, Muzzafar et al. [21] reported in the same year an average measured dose of 0.09 mSv for an intramedullary nail osteosynthesis in the femur, with a mean fluoroscopic time of 233 seconds. According to the new limit, this would allow a maximum of 220 operations of this kind per year.

In 2010, König et al. [12] published results of a prospective study measuring and evaluating the radiation exposure of eyes, hands and legs for three surgeons, individually for each of them using intraoperative radiographic imaging techniques. A comparatively high level of radiation exposure to the eyes was found in forearm, ankle and knee joint operations, with the eye lens dose being sometimes twice that of the hand [12].

Kim et al. [7] and Kesavachandran et al. [14] came to a similar conclusion regarding the large variation of the measured radiation doses.

Strohmaier et al. [23] showed in 2017 that no correlation can be established between the measured dose of the lens dose and the applied dose area product. Many other influencing factors (position of the personnel during the procedure, height, experience, the patient as a scattering body, etc.) are so interlocked here that the estimation of the eye lens dose on simple correlation is not possible. The study also showed that the exposed radiation goggles were not used due to the weight and impracticable handling of the subjects—with a few exceptions [23]. It is therefore recommended in the medical field to sensitize the medical staff regarding the radiation exposure. Self-critically, the authors describe that in orthopedics and trauma surgery, a significant portion of medical staff using perioperative imaging techniques has not been considered [23].

Against this background, the question arises as to how far the future dose limit will be adhered to trauma surgery in the day-to-day work of the surgical staff, or whether greater emphasis on preventive measures may be required.

## 2. Materials and Methods

Between February and October 2018, five trauma surgeons, five hand surgeons and five surgical assistants of the BG Klinikum Hamburg were included in the prospective study. The study was endorsed by the Ethics Committee of the University of Lübeck (Aktenzeichen 18-069 (11/4/18)) and each subject has agreed in writing to participate.

The measurement of the radiation exposure of the eye lens was carried out with personalized partial body dosimeters Hp (0.07) of the brand LPS-TLD-TD 07 (Landesanstalt für Personendosimetrie und Strahlenschutzausbildung, Berlin, Germany). The nominal range is 12 keV- 1250 keV. The evaluation is carried out by the automatic TLD (thermoluminescent dosimeter) reader (model Thermo Scientific HARSHAW 6600plus CCD, Thermo Fisher Scientific Inc., Waltham, Massachusetts, USA).

The contribution to the measurement uncertainty due to the energy and angular dependence of the response is taken into account. At the measuring point (National Institute for Personal Dosimetry and Radiation Protection Training, Berlin, Germany) five reference dosimeters per participant group were used to determine the zero effect.

**Figure 1:**
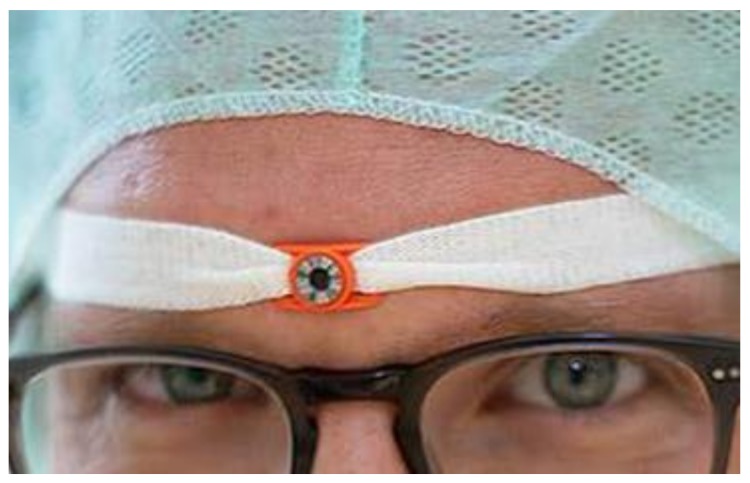
Way of wearing the eye dosimeter.

The dosimeter was fixed with a headband and worn above the eyes (Figure 1). The participants of the study carried the dosimeter constantly during their time in the operating area. The loading and unloading and storage of the dosimeter took place in the operating room. One reference dosimeter was deposited for each group at the repository (staff member changing room) and left there for the entire period of three months.

The personal protective equipment (PPE) of the participants consisted of an X-ray apron and a thyroid protection. The study participants wore no radiation goggles. Fluoroscan InSight2 Mini C-arm (Hologic, Marlborough, Massachusetts, USA) and Philips BV Endura mobile C-arm devices (Philips, Hamburg, Germany) were used as X-ray devices. The dosimeters were sent to the LPS measuring institute for evaluation after three months.

One participant of the study each from the groups of hand surgeons and the surgical assistants lost the dosimeter, so data from five trauma surgeons, four hand surgeons and four surgical assistants could be evaluated. The loss of the two dosimeters was due to the small size and low weight of the dosimeter; two participants of the study threw away the dosimeter by mistake unnoticed when removing the surgical cap.

The duration of surgery, function (assistant, surgeon, surgical assistant), surgical type (procedure, diagnosis), designation of the X-ray system, total duration of fluoroscopy per operating room and dose area product per operating room were recorded from the hospital’s information system.

The dose area product (DAP) (unit Gy × cm^2^) is a measure of dosimetry and basis for calculating the radiation exposure during an X-ray with an X-ray device (e.g. fluoroscopy, mobile C-arm, etc.) and corresponds to the product of dose and area irradiated. The absorbed dose is a physical quantity that indicates the energy imparted by ionizing radiation per unit of mass (unit Gy), whereby the different biological effects of the different types of radiation are not taken into account. The Gray (ɡɹɛɪ̯) (unit symbol Gy) is a quantity derived from the International System of Units (SI) units Joule (J) and Kilogram (kg). It indicates the absorbed dose caused by ionizing radiation and describes the energy absorbed per mass. The unit is the quotient of the absorbed energy and the mass of the body. 1 Gy = 1 J/kg. In order to better compare the absorbed dose of different types of radiation with regard to the harmful effects on organisms, it is multiplied by a weighting factor, the radiation weighting factor. It takes into account the relative biological effectiveness of the radiation. Since the radiation weighting factor is a dimensionless number, the equivalent dose and the absorbed dose have the same dimension. However, to emphasize the difference between the dose sizes for practical use and since the numerical value of the dose size may also change due to the radiation weighting factor, Gray is used for energy doses, and Sievert for weighted dose sizes. For photons, in general, 1 Sievert is equal to 1 Gray, except in cases of low energy, e.g. radiographic imaging (C-arm) used in the study where the photoelectric effect dominates.

Statistics: The statistical analysis of the collected data was done with the software SAS 9.2 (SAS Institute Inc., Cary, NC, USA). From the collected data, mean, standard deviation, median, 1st quartile and 3rd quartile as well as minimum and maximum were calculated descriptively. Boxplots were made using the SAS 9.2 software (SAS Institute Inc., Cary, NC, USA). All other graphs were made using the Microsoft Excel® software (Microsoft, Redmont, USA).

## 3. Results

During the study period, a total of 1100 surgeries were performed by the probands involved, including 655 operations using radiological imaging techniques.

### 3.1. Results—Trauma Surgeons

**Figure 2:**
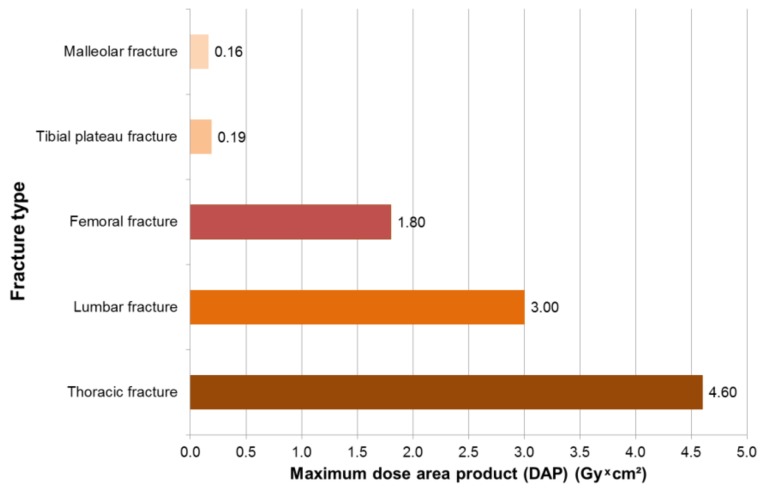
Maximum dose area product for trauma surgeons, according to fracture type.

**Figure 3:**
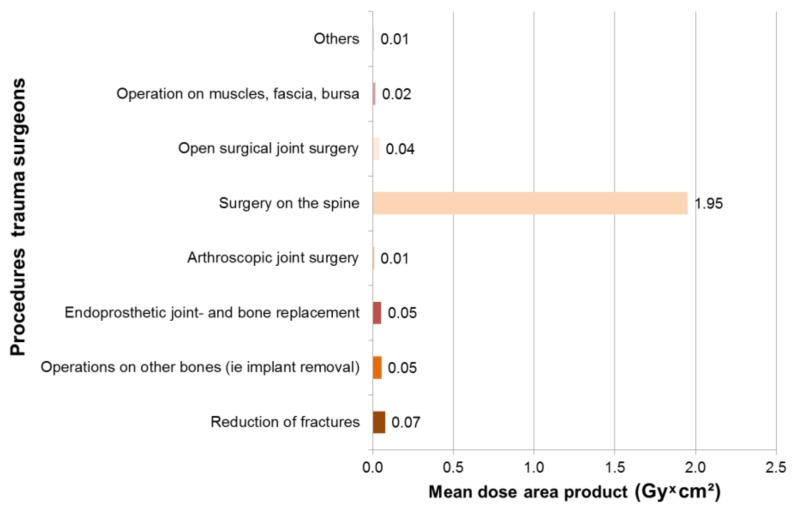
Mean dose area product for trauma surgeons, according to procedure type.

In 249 surgeries with intraoperative X-rays, maximum dose area products occurred for trauma surgeons in thoracic fractures of 4.6 Gy × cm², followed by lumbar fractures of 3 Gy * cm² and femur fractures of 1.8 Gy * cm² (Figure 2). The mean dose area product was 1.95 Gy * cm^2^ for spinal surgery and 0.08 Gy * cm^2^ for fracture reduction (Figure 3). The X-ray devices issue the dose area product with the fourth decimal place. This is not necessary for the reader, so we have limited the values to two decimal places.

### 3.2. Results—Hand Surgeons

**Figure 4:**
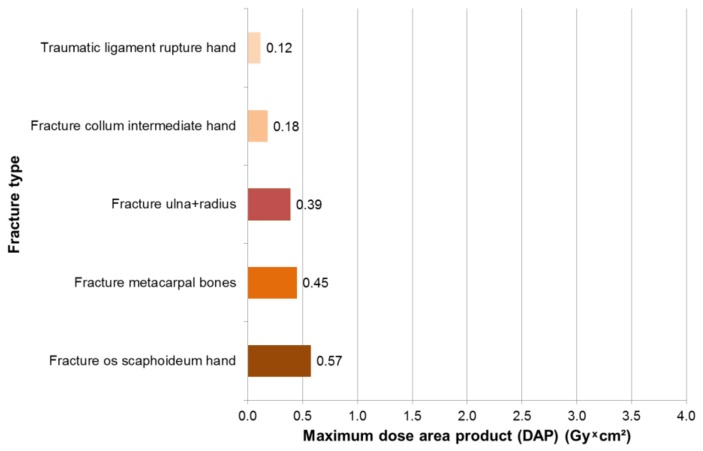
Maximum dose area product for hand surgeons, according to fracture type.

**Figure 5:**
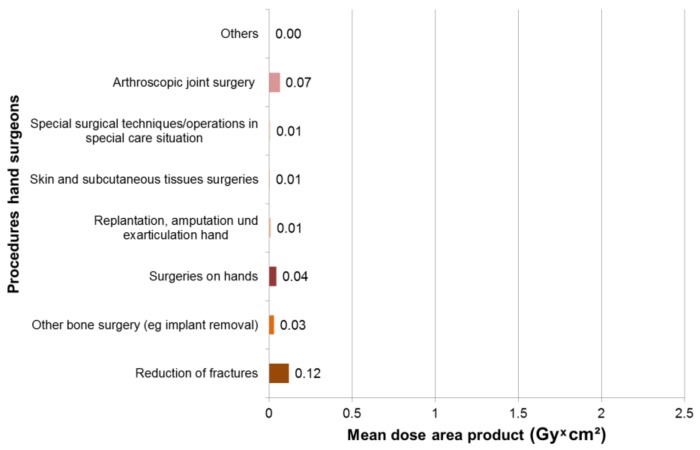
Mean dose area product for hand surgeons, according to procedure type.

In 169 surgeries with intraoperative X-rays, maximum dose area products occurred for hand surgeons in fractures of the Os scaphoid with 0.57 Gy × cm², followed by 0.45 Gy × cm² fractures of the metacarpals and 0.39 Gy × cm² fractures of the ulna and radius (Figure 4). Most procedures with intraoperative X-ray diagnostics were reduction of fractures (*n* = 76), operations on other bones (*n* = 40) and manual operations (*n* = 30). The mean dose area product was 0.12 Gy × cm² for fracture reduction, 0.07 Gy × cm^2^ for arthroscopic joint surgery and 0.04 for manual surgery Gy × cm² (Figure 5).

### 3.3. Results Surgical Assistants

**Figure 6:**
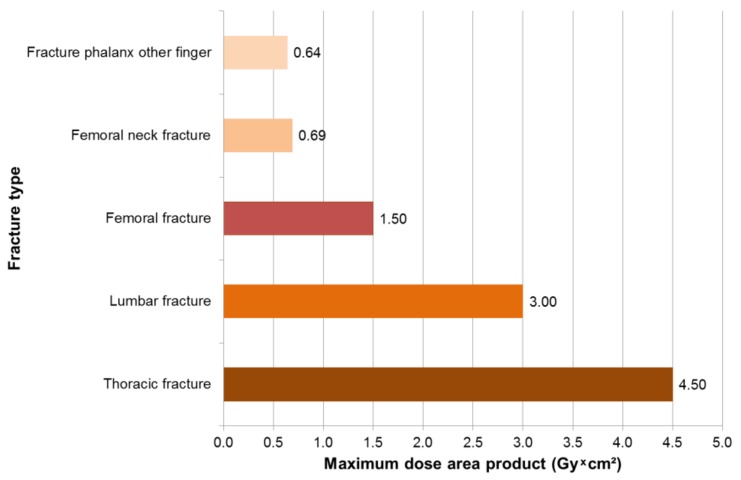
Maximum dose area product for surgical assistants, according to fracture type.

**Figure 7:**
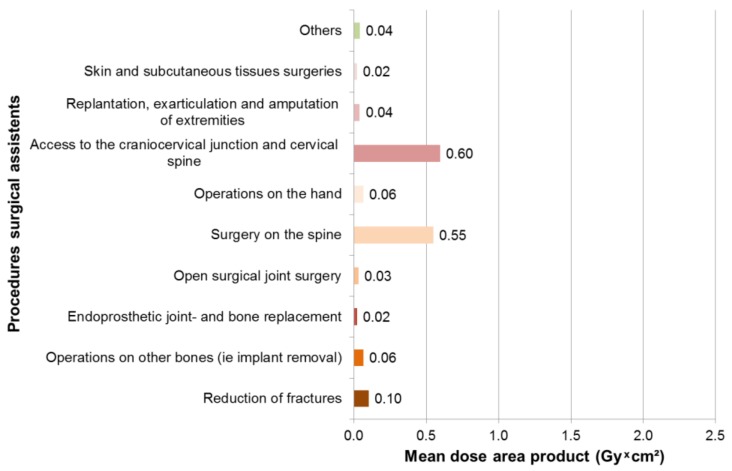
Mean dose area product for surgical assistants, according to procedure type.

In 237 surgeries with intraoperative X-rays, maximum dose area products occurred for surgical assistants in 4.5 Gy × cm² in thoracic fractures, followed by lumbar fractures of 3 Gy × cm² and femur fractures of 1.5 Gy × cm² (Figure 6). The mean dose area product was 0.6 Gy × cm² for surgery on the spine, 0.6 Gy × cm² for access to craniocervical junction and cervical spine surgery and 0.1 Gy × cm² for fracture reduction (Figure 7).

### 3.4. Comparison of Results

Table 1 shows the statistical characteristics of fluoroscopy time, dose area product and duration of surgery, in which intraoperative X-ray was performed.

**Figure 8:**
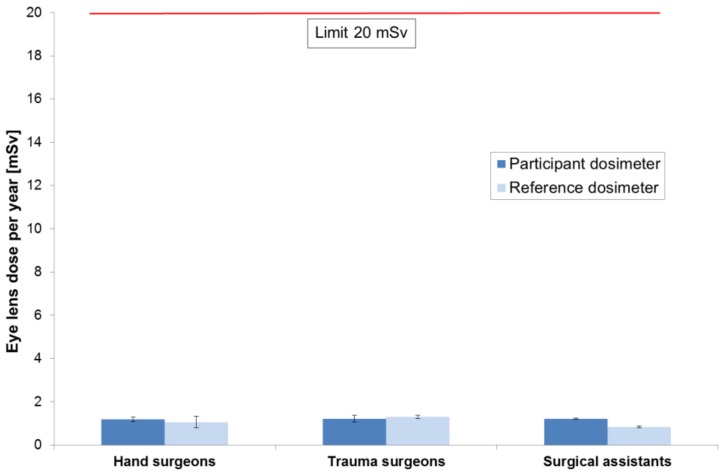
The mean of eye lens dose of the three participant groups (mid-blue) before deduction of the zero effect (measuring point measuring institute LPS) each compared to the mean and standard deviation of the reference dosimeter dose (light blue). The graphic is scaled to the eye lens dose limit of 20 mSv (red line). The values are extrapolated to 1 year.

Both the evaluation of the dosimeters by the trauma surgeons and the evaluation of the dosimeters by the hand surgeons and the surgical assistants revealed no significant radiation exposure of the eye lens in comparison to the respective measured reference dosimeters (Figure 8, Table 2).

The evaluated reference dosimeters, that were in the surgeons changing room during the three months, showed the same measured values as the measured zero effect at the measuring point LPS (National Institute for Personal Dosimetry and Radiation Protection Training, Berlin, Germany). The zero effect represents the measured natural radiation exposure. After subtracting the measured zero effect (measuring point measuring institute LPS), very low to negligible net values with respect to the radiation exposure of the eye lens were found, with the highest load of 0.1 mSV ± 0.03 in the surgical assistants. The hand surgeons’ reference dosimeter showed a value of 0.266 ± 0.065 mSv over the three-month measurement period, so 1.065 mSv are assumed when extrapolated for the year. The trauma surgeons’ reference dosimeter showed a value of 0.323 ± 0.02 mSv over the three-month measurement period, so 1.292 mSv are assumed when extrapolated for the year. The surgical assistants’ reference dosimeter showed a value of 0.209 ± 0.011 mSv over the three-month measurement period, so 0.836 mSv are assumed when extrapolated for the year. Because of the fact that the duration of the measurement period is different for all three groups (the time from the erasure of the dosimeter to the readout), the three zero effects of our reference dosimeters differ.

## 4. Discussion

Intraoperative X-ray diagnostics were used in 655 operations of our study. The fluoroscopy time varied on average between 19 and 49 seconds. In addition, maximum dose area products were found to be 4.6 Gy * cm² in thoracic fractures, followed by lumbar fractures of 3 Gy * cm² and femur fractures of 1.8 Gy * cm². Despite the fluoroscopy time and the sometimes-high radiation dose administered, it was not possible to detect an increased radiation dose to the eye lens compared to the reference dosimeters. This suggests that the individual behaviors of the surgeons and surgical assistants of the BG Klinikum Hamburg (hospital of The Hospital Group of the Statutory Accident Insurance, Berlin, Germany) are efficient with regard to compliance with the radiation protection measures. Limiting to the study are certainly the small number of study participants and the short examination period of only three months. Due to the diversity of the examined surgeries covering the entire spectrum of orthopedics, trauma surgery and hand surgery, a standardized description of the operating room and operating behavior is not possible. Depending on the surgical procedure (microscopic hand surgery, trauma spine surgery etc.), the surgeon’s position relative to the radiation source as well as the viewing direction during image acquisition always changed. Also, a standardized distance between the operator and imager cannot be specified, which is also due to the diversity of the examined operations and the differences between the specializations of the surgeons. Since there is no interventional cardiology, radiology or gastroenterology in our facility, to which the surgical assistants involved in the study could be assigned, the study is only transferable to trauma hospitals with the same spectrum of operations.

Medical personnel, specifically operating personnel who are exposed to perioperative X-rays, as the literature shows, are subject to a significantly increased risk of radiation-induced cataracts. For this reason, the limit value for the eye lens in 2018 has been drastically reduced from 150 mSv/year to 20 mSv/year [3]. Strohmaier et al. showed in 2017 that there is neither a correlation between the measured dose of the eye lens nor the applied dose area product (in Gy) nor the number of interventions [23].

Investigations at the beginning of the use of intraoperative X-ray diagnostics (non-radiologist imaging) have shown that high doses of radiation are delivered to the patient and medical staff so that immediate or long-term radiogenic effects can be caused [8,20].

Since the introduction of the first C-arm in 1955, not only the technology has evolved rapidly. Through automatic dose rate control, dose rate limitation, pulsed fluoroscopy, improved detection technology with effective beam limitation on image intensifier diameter and digital image processing, the applied dose has been significantly reduced in recent years with improved image quality. At the same time, more attention has been paid to the topic of radiation protection, especially in the case of operationally active personnel, at the beginning of training (specialist in radiation protection “Fachkunde Strahlenschutz”). In radiation protection, structural measures can be distinguished from apparatus-related and personnel-operational measures. The use of classical C-arms in the operating room presents more difficult conditions in terms of radiation protection compared to the fixed radioscopy units: little own shielding, no spatially limited control area, no constructional protective measures for the OR (Operating Room) staff, little distance between staff member and patient, often above table arrangement of the radiation source and often long fluoroscopy times. Therefore, in particular the personnel-operational protective measures have been optimized as a focus for the prevention of radiation damage. In terms of fluoroscopy time, the use of interval-pulse fluoroscopy and load-image-hold technology can be significantly reduced perioperatively. In addition, the laser aiming device of modern C-arms greatly reduces orientation radiation.

Various studies have shown that in addition to a minimization of fluoroscopy time, a large distance to the radiation source is the most effective protection, since a further decisive point of radiation protection is compliance with the inversed square law: The dose is reduced quadratically with the distance from the radiation source.

For a long time, the over-table position of the radiation source in the operating room was considered to be the better, especially as it ensured the surgeon’s working height. Modern new flat-panel image detectors can be significantly space-saving and thus solve the problem of working height at under-table position. But not only the anterior-posterior orientation of the C-arm plays a crucial role—the positioning in the lateral beam path is also a key factor. Rampersaud et al. evaluated that the dose to the torso of the surgeon may be very high in spine surgery with lateral orientation of the C-arm, particularly on the side of the X-ray source. On the detector side, i.e. in the direction of radiation, the dose area product to the torso of the surgeon is much lower [48].

Surgery on the spine, with a mean dose area product of 1.95 Gy * cm² per procedure, is by far the highest radiation exposure for our hospital. This has also been described in previous studies [8,9,10,11,12,13]. A 2005 study by Harstall et al. [21] reported an equivalent dose of 11.9 mSv per year for 2 spinal surgeons, who had performed 32 vertebroplasties together during this period. This would correspond to approximately 60% of the new limit of 20 mSv/year. As another limiting factor of our study, it should be noted that, despite the high number of evaluated surgeries using intraoperatively native radiographic imaging techniques, the spinal interventions represent only a relatively small percentage (3%). This is certainly due to the operational portfolio of our company as a maximum-care, trans-regional trauma center. For smaller clinics, where the percentage of age trauma or elective minimally invasive spine surgery is significantly higher, the radiation exposure of surgeons and surgical assistants be significantly increased. Therefore, the strict compliance with the radiation protection measures should be even more important for these departments. Here it would certainly be useful in a further investigation to firmly investigate the radiation exposure of the lens in spine surgery.

## 5. Conclusions

In conclusion, it can be stated that mainly the individual radiation protection measures of the participants of the study have led to a reduction of the measured eye lens dose to an absolute minimum. The studied participants represent a mixed collective that can be transferred to other trauma hospitals that provide emergency care. It shows that the new limit value of 20 mSv/year is clearly not being met and that the new limit of 20 mSv/year does not require any additional action. Because the risk of cataract formation cannot be ruled out in principle, wearing X-ray goggles should nevertheless be considered as worthwhile.

A limitation could be given in special cases, however; surgical interventions on the spine play a crucial role here. In conclusion, it can thus be stated that the new (reduced) limit value of 20 mSv/year for the surgeon and his surgical team is complied with, provided that the aforementioned radiation protection measures are considered and applied.

## Figures and Tables

**Table 1:** Statistical characteristics of fluoroscopy time, dose area product and duration of surgery.

	Surgeries with Intraoperative X-Rays		
Statistical Characteristics	Trauma Surgeons (*n* = 249)	Hand Surgeons (*n* = 169)	Surgical Assistants (*n* = 237)
	Duration of radioscopy (Sec)		
Median	8	29	11
Quartil 1	3	9	4
Quartil 3	21	63	36
Minimum	1	1	1
Maximum	230	305	407
	Dose area product (Gy × cm^2^)		
Median	0.02	0.03	0.02
Quartil 1	0.01	0.01	0.01
Quartil 3	0.04	0.08	0.08
Minimum	<0.00	<0.00	<0.00
Maximum	4.6	0.57	4.47
	Duration of the surgery (Min)		
Median	59	70	69
Quartil 1	38	42	42
Quartil 3	81	119	102
Minimum	10	5	10
Maximum	229	512	638

**Table 2:** Annual radiation exposure.

	Hand Surgeons		Trauma Surgeons		Surgical Assistants	
Statistical Characteristics	Participants (mSv) *n* = 4	Reference (mSv) *n* = 5	Participants (mSv) *n* = 5	Reference (mSv) *n* = 5	Participants (mSv) *n* = 4	Reference (mSv) *n* = 5
Mean	1.20	1.06	1.22	1.29	1.22	0.84
Standard deviation	0.10	0.26	0.16	0.08	0.04	0.04
